# Understanding the role of trehalose in interactions between *Wolbachia* and *Aedes aegypti*

**DOI:** 10.3389/fcimb.2025.1547873

**Published:** 2025-03-18

**Authors:** Benjamin Dupuis, Nicolas Pocquet, Anna-Bella Failloux

**Affiliations:** ^1^ Institut Pasteur, Université Paris Cité, Arboviruses and Insect Vectors, Paris, France; ^2^ Institut Pasteur de Nouvelle-Calédonie, Unité de Recherche et d'Expertise en Entomologie Médicale (URE-EM), Nouméa, New Caledonia

**Keywords:** trehalose, *Wolbachia*, pathogen-blocking effect, *Aedes aegypti*, antioxidant, autophagy

## Abstract

Mosquito-borne diseases such as chikungunya, dengue, and Zika represent a major burden on global public health. To fight against these arboviruses, vector control strategies are a priority. One existing strategy is based on the use of an endosymbiotic bacterium, *Wolbachia*, which reduces the transmission of arboviruses by the mosquito *Aedes aegypti via* a pathogen blocking effect. *Wolbachia* in *Ae*. *aegypti* disrupts several pathways of the host’s metabolism. Trehalose is a carbohydrate circulating mainly in insect hemolymph and plays a role in numerous mechanisms as energy source or stress recovery molecule and in chitin synthesis. This study explores the importance of trehalose in the interactions between *Wolbachia* and *Ae. aegypti*, and attempts to understand the pathogen blocking effect.

## 1 Introduction

Vector-borne diseases exert a significant impact on public health contributing to global morbidity and mortality (Bhatt et al., 2013; LaBeaud et al., 2011). For example, 400 million people are infected by dengue virus (DENV) in the world each year (Bhatt et al., 2013; WHO, 2023a), costing $9 billion (Shepard et al., 2016). *Aedes aegypti* is a primary vector of several arboviruses including DENV (WHO, 2023b). There is no specific treatment against dengue fever and vector control remains the main strategy to limit its emergence and rapid spread. Among vector control strategies (Achee et al., 2019), insecticide treatments are mainly implemented to interrupt dengue transmission (Van Den Berg et al., 2021). However, control efforts have failed to limit dengue fever epidemics and targeted mosquito populations developed resistance to insecticides (Asgarian et al., 2023), urging the development of alternative vector control strategies. Among them, vector control using *Wolbachia pipientis* has met with significant success (Hilgenfeld and Vasudevan, 2018).

*Wolbachia* are Gram-negative bacteria belonging to the *Alphaproteobacteria* class and the *Rickettsiales* order. Discovered in 1924 (Hertig and Wolbach, 1924), *Wolbachia* are obligate intracellular bacteria found in nematodes, insects and other arthropods; 40-60% of insects are infected by *Wolbachia* (Weinert et al., 2015). These bacteria are vertically transmitted and manipulate their hosts to secure their own transmission to the progeny by: (i) inducing a sex ratio distortion in favor of *Wolbachia*-infected females *via* parthenogenesis, feminization, male killing or (ii) sterilizing certain individuals *via* cytoplasmic incompatibility (Landmann, 2019). These effects are not shared by all *Wolbachia* strains, e.g. *Wolbachia* from filarial nematodes. In arthropods, *Wolbachia* strains manipulate the host reproduction and inhibit the transmission of some pathogens, making them potential candidates for vector control (Gill et al., 2014). In insects, *Wolbachia* is mainly present in reproductive tissues (ovaries) but also in somatic tissues such as the midgut, fat body, salivary glands and muscles (Mejia et al., 2022; Zouache et al., 2009). The relationship between the host and the endosymbiont can be parasitic, mutualistic or commensal (Newton and Rice, 2020), depending on cellular host conditions and the availability of certain nutrients (Lindsey et al., 2018). In addition, the bacterium modifies the host intracellular environment (Lindsey et al., 2018) affecting the cytoskeleton (microtubule and actin) (Ferree et al., 2005; Sheehan et al., 2016) and also modulates gene expression in the host cell; differential expression of genes related to metabolism (Molloy et al., 2016), immunity (Xi et al., 2008) or synthesis of antioxidant molecules [21, 22]. Additionally, *Wolbachia* induce oxidative stress (Pan et al., 2012), which indirectly affects host metabolism (Bhardwaj and He, 2020) and immunity (Pan et al., 2012; Zug and Hammerstein, 2015). All these changes induced by *Wolbachia* affect the replication and transmission of DENV in *Ae. aegypti* (Reyes et al., 2021; Terradas and McGraw, 2017). This phenomenon is known as pathogen blocking effect or pathogen interference. The exact mechanisms leading to the pathogen-blocking effect are not yet fully elucidated, but they appear to be multifactorial, involving immunity, competition for resources, lipids, and oxidative stress (Reyes et al., 2021; Terradas and McGraw, 2017).

*Wolbachia* strategy is at the base of two distinct and complementary approaches in vector control: (i) incompatible insect technique based on cytoplasmic incompatibility to reduce the size of vector populations and (ii) pathogen-blocking effect to limit the transmission of arboviruses (Caragata et al., 2021). The most commonly *Wolbachia* strains used for vector control are *w*Mel from *Drosophila melanogaster* and *w*AlbB from *Aedes albopictus* trans-infected in *Ae. aegypti* (Caragata et al., 2021; Ross, 2021). The pathogen blocking phenotype is *Wolbachia* strain- and host-dependent (Jiménez et al., 2019); *w*AlbB enhances, rather than inhibits, West Nile virus infections in *Culex tarsalis* (Dodson et al., 2014; Glaser and Meola, 2010). The effects produced by *Wolbachia* are based on a variety of cellular and molecular mechanisms. Indeed, *Wolbachia* have an effect on the carbohydrate and lipid metabolism and among carbohydrates, trehalose is an essential molecule for growth, fertility, and vitality.

**Figure 1:**
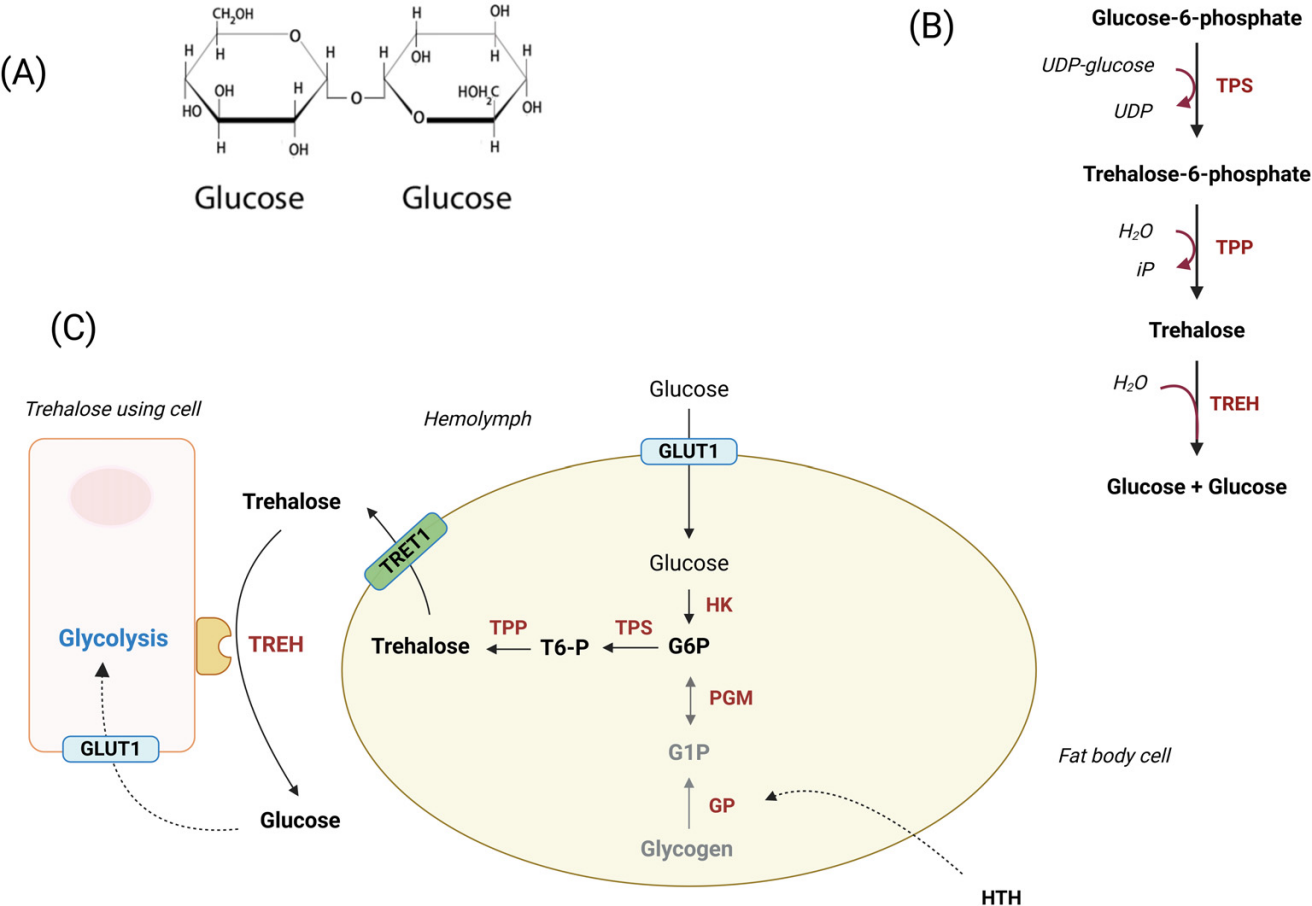
Trehalose molecule, synthesis and pathway. **(A)** Haworth representation of trehalose, a glucose dimer linked by a α,α-1,1 bond. **(B)** Trehaloneogenesis, the trehalose-forming metabolic pathway. **(C)** Trehalose involved in trehaloneogenesis and glycolysis. UDP, Uridine diphosphate glucose; iP, inorganic phosphate; TPS, Trehalose 6-phosphate synthase; TPP, Trehalose 6-phosphatase; TREH, Trehalase; GLUT1, Glucose transporter I; HK, Hexokinase; PGM, Phosphoglucomutase; GP, Glycogen phosphorylase; G1P, Glucose 1-phsophate; G6P, glucose 6-phosphate; T6P, trehalose 6-phosphate; HTH, Hypertrehalosemic hormone;TRET1, Trehalose transporter I (Created in https://BioRender.com).

Trehalose (α-D-glucopyranosyl-α-D-glucopyranoside) is a non-reducing disaccharide found in insects (**Figure 1A**). Present in soluble form at high concentrations (5 to 50mM) in insect hemolymph, its concentration is highly dependent on environmental conditions, nutrition and the insect state of stress (homeostasis disturbance) (Becker et al., 1996; Tamayo et al., 2022; Thompson, 2003). Trehalose participates in several physiological processes such as metabolism, development, chitin synthesis, flight, recovery from stress, and more globally in maintaining homeostasis (Matsuda et al., 2015; Shukla et al., 2015).

We examine the role of trehalose in interactions between *Wolbachia* and an arbovirus in *Ae*. *aegypti*, and discuss implications of these relationships for the control of mosquito-borne diseases.

## 2 Trehalose in *Aedes aegypti*

Trehalose is a molecule synthesized in the fat body through trehaloneogenesis from glucose acquired with food. The trehaloneogenesis pathway diverts one of the intermediates of glycolysis: glucose-6-phosphate (G6P) as substrate for trehalose-6-phosphate synthase (TPS) producing trehalose-6-phosphate (T6P) which is dephosphorylated *via* trehalose-6-phosphate-phosphatase (TPP) giving trehalose (**Figure 1B**) (Shukla et al., 2015). Then, this molecule is released in the hemolymph *via* the trehalose transporters TRET1 (Tamayo et al., 2022) and cleaved into two glucose molecules by trehalases (TREH), which operate in two forms: TREH-1 soluble and TREH-2 anchored in the membrane of all insect cell types (muscle, midgut, salivary glands, fat body) (Tamayo et al., 2022). Finally, glucose molecules produced by cleavage are imported into cells *via* glucose transporters (GLUT1), initiating multiple metabolic pathways including glycolysis (**Figure 1C**) (Tamayo et al., 2022). Trehalose can also be imported into various types of cells *via* TRET1 or through active transporters. Indeed, TRET1 is highly expressed in fat body cells and muscles (Kanamori et al., 2010) while Malpighian tubules possess active transporters for trehalose (**Figure 1C**) (Kanamori et al., 2010).

Trehalose plays a fundamental role in insects due to its versatility in many physiological processes (Thompson, 2003). As the main circulating carbohydrate, it serves not only as an energy source for metabolic activities (Becker et al., 1996; Shukla et al., 2015; Thompson, 2003), but is also involved in energy storage in the form of glycogen (Zeng et al., 2020). Additionally, trehalose acts as a protector against extreme environmental conditions, providing cryoprotection against cold (Huang et al., 2022) and shielding cells from desiccation (Thorat et al., 2012). It is also involved in chitin synthesis (Yang et al., 2024), an essential component of insect exoskeleton, and plays a role in key biological processes such as oogenesis and diapause (Becker et al., 1996; Zeng et al., 2020). Finally, trehalose is crucial for recovery after periods of stress, demonstrating its vital importance for insect survival and adaptation to changing environments (Shukla et al., 2015; Thompson, 2003; Yasugi et al., 2017; Yu et al., 2020).

In insects, trehaloneogenesis is a pathway regulated by hormones (Tellis et al., 2023; Thompson, 2003). Indeed, trehaloneogenesis is activated in fat body cells by hormones secreted from the nervous system (e.g. juvenile hormone analogs, 20-Hydroxyecdysone (20E) and diuretic hormone (DH)) (Shukla et al., 2015; Tellis et al., 2023). Some factors such as exposure to pesticides and temperature shifts, increase trehalase activity while others (hormonal regulation *via* regulating factors) reduce it (Shukla et al., 2015; Tellis et al., 2023). Thus, trehalose concentration varies throughout insect life. The hypertrehalosemic hormone (HTH) is responsible for regulating trehalose levels in insects (Thompson, 2003). HTH is primarily released in response to increased energy demand or stressful conditions, and it stimulates the release of stored trehalose from tissues to provide an additional energy source for the insect (Thompson, 2003). This helps to maintain trehalose homeostasis and ensures that insects have a readily available energy source when needed (Tellis et al., 2023); HTH is released when insects need additional energy (e.g. for flight) (Thompson, 2003). This stimulates the breakdown of glycogen stored in tissues into glucose (Thompson, 2003). Subsequently, glucose is converted into trehalose, which is released into the hemolymph (Tellis et al., 2023; Thompson, 2003). Additionally, the regulation of trehalose concentration in the hemolymph is ensured by modulating the expression of trehalose transporters, such as TRET1, in the fat body cells (Kanamori et al., 2010; Tamayo et al., 2022).

## 3 Trehalose as an energy source for *Wolbachia* in *Ae. Aegypti*

The presence of *Wolbachia* disrupts various cellular dynamics of its host. Indeed, *Wolbachia* can modify the expression of certain host genes (Caragata et al., 2017; Lindsey et al., 2021). Genes involved in membrane transport (glucose transporter, permeases, monocarboxylate or cholesterol transporters) and carbohydrate metabolism are over-expressed, activating the host cell global metabolism (Caragata et al., 2017). Induced changes increase the amount of nutrients imported and metabolic activity, as well as trehalose concentration which depends on iron availability in the organism (Currin-Ross et al., 2021). However, it has been shown that *Wolbachia* can use trehalose and glycogen reserves, increasing the amount of glucose (Zhang et al., 2021). *Wolbachia w*Mel strain lacks a trehalose-forming pathway, but synthesizes enzymes that can import and use trehalose (Jiménez et al., 2019). Thus, the *Wolbachia-*infected host may use trehalose to obtain glucose, a main source of energy (Currin-Ross et al., 2021), as well as other nutrients such as glyceraldehyde 3-phosphate (GP3) (Jiménez et al., 2019; Lindsey et al., 2018). The bacterium induces an increased level of glucose (Zhang et al., 2021) and therefore participates in energy metabolism such as glycolysis (Shukla et al., 2015), providing it with essential metabolic intermediates and precursors (G3P) and energy. NADH synthetized could indirectly benefit *Wolbachia via* the pentose phosphate pathway using G6P as a metabolic precursor.

*Wolbachia* also affects host hormone regulation. Indeed, this bacterium impacts the regulation of its host’s insulin/IGF-like signaling pathways (Ikeya et al., 2009). The insulin/IGF pathway, in turn, is known to affect trehalose carbohydrate storage in insects (Bobrovskikh and Gruntenko, 2023; Broughton et al., 2005). Furthermore, it has been shown that the level of juvenile hormone (JH) is elevated in *Wolbachia*-infected *Drosophila melanogaster* males (Zhang et al., 2021). This hormone is known to regulate trehalose synthesis (Xu et al., 2013). Finally, *Wolbachia*-induced oxidative stress (Pan et al., 2012) leads to a hormonal response with synthesis of hormones belonging to the adipokinetic hormones (AKH) family (Chaitanya et al., 2016); this hormone also plays a role in increasing the amount of trehalose, using glycogen reserves for energy purposes (Huang et al., 2012; Lu et al., 2019). The increased amount of energy produced would compensate for losses of resources diverted by *Wolbachia*. Glycolysis is an essential component of intracellular bacteria-host interactions; it was shown that pyruvate maintains the symbiotic relationship (Melnikow et al., 2013; Voronin et al., 2019, 2016). In addition, in immune cells, sugar metabolism (from trehalose and glucose), particularly the pentose phosphate pathway (PPP cycle), is crucial not only for fighting and regulating infections but also for protecting the host from the effects of its own immune response and for ensuring the fitness (Kazek et al., 2024). This phenomenon could be accentuated with DENV infection. Indeed, competition for resources and energy becomes critical, and trehalose regulation may help in host cell survival.

## 4 Trehalose as a key molecule in the regulation of oxidative stress

As previously mentioned, *Wolbachia* induces oxidative stress, resulting in increased concentrations of reactive oxygen species (ROS) (H_2_O_2_, O_2_•, OH•) (Pan et al., 2012). Oxidative stress is unsustainable for the cell if prolonged, as ROS induce lipid peroxidation, damage to genetic material or apoptosis (Kodrík et al., 2015; Martemucci et al., 2022). To preserve cell integrity, it is vital to counterbalance this oxidative stress with antioxidant mechanisms (Felton and Summers, 1995). These can be antioxidant enzymes such as Glutathione S-transferase (GST), or antioxidant molecules such as glutathione, ascorbic acid, uric acid and carbohydrates (Felton and Summers, 1995), which are ubiquitous in insect tissues (fat bodies, midgut, Malpighian tubes) (Felton and Duffey, 1992). Antioxidant molecules trap molecules responsible for oxidative stress (Felton and Summers, 1995). As trehalose is a carbohydrate, it could be a key molecule in the regulation of *Wolbachia*-induced oxidative stress in *Ae. aegypti*. Indeed, trehalose can scavenge free radicals, thus acting as an antioxidant, at least in insect hemolymph (Felton and Summers, 1995). Thus, oxidative stress can be attenuated by regulating trehalose metabolism (Peng et al., 2024; Thorat et al., 2016). Similarly, trehalose intake reduces the amount of oxidizing molecules, giving it at least an antioxidant effect (Peng et al., 2024). The increase in trehalose seems to be involved in regulating redox balance (Thorat et al., 2016). Furthermore, trehalose is also involved in redox balance in many other organisms (antioxidant molecule), as in yeast, certain bacteria, mammals and plants (Benaroudj et al., 2001; Luo et al., 2008; Reyes-DelaTorre et al., 2012; Zhang et al., 2023), trehalose accumulation during cellular stress such as oxidative stress may reduce free radical damage *via* mechanisms that remain unclear (Benaroudj et al., 2001; Reyes-DelaTorre et al., 2012). We therefore suggest a similar accumulation mechanism in *Ae. aegypti*, participating in the regulation of oxidative stress, as *Wolbachia* does (Pan et al., 2012).

## 5 Trehalose in *Ae. aegypti - Wolbachia* interactions

Interactions between *Wolbachia* and *Ae. aegypti* can take various forms and lead to different effects: metabolic (Lindsey et al., 2018; Ponton et al., 2015; Reyes et al., 2021), immune (Caragata et al., 2017; McGraw, 2004; Souza-Neto et al., 2009; Ye et al., 2013) or others (Lindsey et al., 2018). All together they impact DENV replication, leading to a pathogen-blocking effect (Reyes et al., 2021; Terradas and McGraw, 2017). Exchanges and interactions between *Wolbachia* and its mosquito host take place *via* diverse detection and signaling processes (Lindsey, 2020). In addition, *Wolbachia* interacts with its host’s RNA (Terradas et al., 2017); the miRNA (micro) pathway participates in “pathogen-blocking” effect by *w*MelPop strain in *Ae. aegypti* (Hedges et al., 2008). Inhibition of certain miRNAs leads to a decrease in *Wolbachia* density, suggesting that the endosymbiont facilitates its maintenance in the host by manipulating host gene expression *via* miRNAs (Zhang et al., 2013), as piRNAs (Mayoral et al., 2014). piRNAs are important in cell signaling and host immune responses. Nevertheless, the nature of all the interactions between *Wolbachia* and its host is not fully understood.

### 5.1 Trehalose as potential glycolipid

Trehalose may play a role in these interactions. This molecule has been shown to be crucial in interactions between the bean bug and a Gram-negative symbiont bacterium (Lee et al., 2023). The same role can be suggested in the relationship between *Ae. aegypti* and *Wolbachia*. Indeed, the trehalose imported by the cell carrying *Wolbachia* could directly serve the endosymbiont itself. It is believed that *Wolbachia* do not have their own trehaloneogenesis pathway (Jiménez et al., 2019). However, the *Wolbachia w*Mel strain possesses a PEP (phosphoenolpyruvate) system for trehalose transport within the bacterium (Jiménez et al., 2019), as well as a trehalose 6-phosphate-specific phosphohydrolase, resulting in trehalose production (Jiménez et al., 2019). A portion of the trehalose could be used for the synthesis of trehalose-forming glycolipids, as it is the case in other types of intracellular bacteria (Asselineau and Asselineau, 1978; Reinink et al., 2019). A hypothesis here is that these glycolipids could play a role in the *Wolbachia*-induced pathogen-blocking effect in *Ae. aegypti*. Indeed, these molecules are reported as having immunostimulant properties (Asselineau and Asselineau, 1978; Vanaporn and Titball, 2020) and can be brought into contact with the host through as yet unknown mechanisms, supporting the immune system priming hypothesis in pathogen blocking effect.

### 5.2 Trehalose as an autophagy inducer

Autophagy is a self-degradative process pivotal for re-equilibrating energy sources at critical times in development and response to stress (Jo et al., 2021; Li et al., 2022; Tracy and Baehrecke, 2013). Autophagy is a highly conserved intracellular mechanism in eukaryotes (Glick et al., 2010; Kuo et al., 2018). Although the mechanism remains universal, some proteins involved in this process are different in insects (Brackney, 2017; Jain et al., 2015; Kuo et al., 2018). Autophagy is an important process involved in interactions between *Wolbachia* and *Ae. aegypti*. Host autophagy directly affects *Wolbachia* density (Deehan et al., 2021), but also *Wolbachia* infection (Hargitai et al., 2022). Autophagy could also indirectly allow *Wolbachia* colonization in *Ae. aegypti*, since this mechanism regulates oxidative stress (Chaitanya et al., 2016; Wu et al., 2009), avoiding apoptosis. Activation of autophagy can also eliminate pathogens such as arboviruses (Jo et al., 2021; Mahanta et al., 2023; Wu et al., 2009), since arboviruses also influence autophagy in infected cells (Barletta et al., 2016; Echavarria-Consuegra et al., 2019; Heaton and Randall, 2011). Trehalose has been shown to induce and regulate autophagy in many organisms, with mechanisms that need to be determined. This molecule induces autophagy leading to various effects in human cells: an antiviral effect (Belzile et al., 2016), an antioxidant effect (Honma et al., 2018; Mizunoe et al., 2018), an induction of apoptosis in the cell (Darabi et al., 2018) or a renewal effect of intracellular components (Xu et al., 2019). Trehalose-inducing autophagy effect is also described in plants (Williams et al., 2015). It is conceivable that the highly conserved process of autophagy could also be induced by trehalose in insect cells and participates in interactions between *Wolbachia* and *Ae. aegypti*.

## 6 Conclusion and discussion

**Figure 2:**
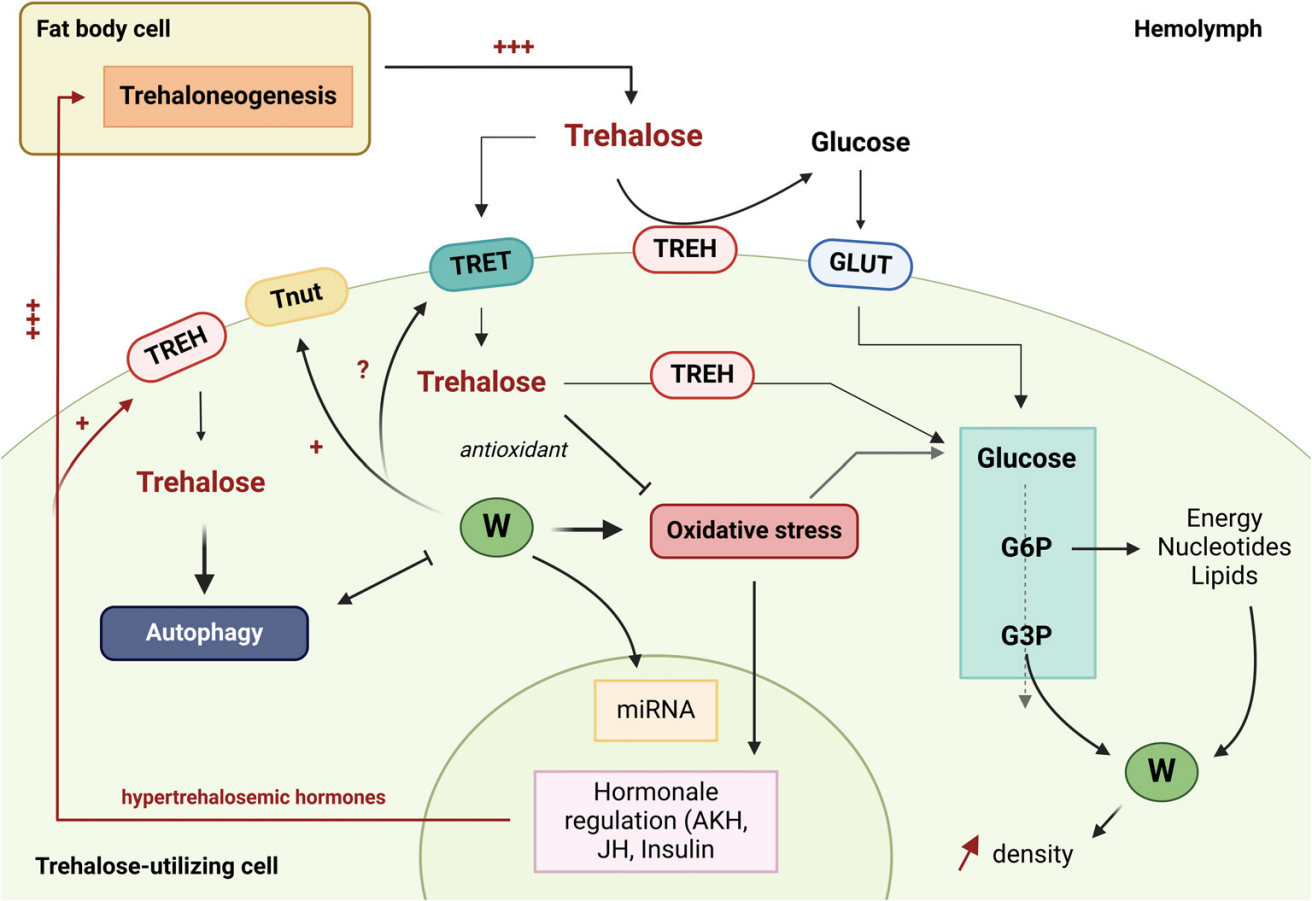
The role of trehalose in interactions between *Wolbachia* and *Aedes aegypti*. The green circles with a W represent *Wolbachia* bacteria. TREH, Trehalase; GLUT1, Glucose transporter; TRET, Trehalose transporter; Tnut, Transporter of nutrients; G6P, glucose 6-phosphate; G3P, Glycéraldéhyde-3-phosphate; JH, Juvenile hormone; AKH, Adipokinetic hormones; miRNA, microRNA. (Created in https://BioRender.com).

Trehalose is an essential disaccharide for insects. Its role in maintaining metabolism in the presence of *Wolbachia*, in stress recovery, as an antioxidant and potentially as an inducer of autophagy and priming of the immune system through glycolipid synthesis, makes it a key molecule to understand the *Ae. aegypti* - *Wolbachia* - arbovirus relationship (**Figure 2**).

Despite the knowledge recently acquired on the interactions between *Wolbachia* and *Ae. aegypti*, many questions remain unresolved. Future research avenues are proposed, including experimental studies to elucidate the precise role of trehalose in *Wolbachia* colonization and pathogen blocking effect. The potential role of trehalose in autophagy induction should be addressed by experimentally testing if trehalose supplementation leads to autophagic structure formation. Similarly, the presence of trehalose forming lipids should be confirmed.

It has been shown that inactivation of TRET decreases significantly trehalose concentration in the hemolymph of *Anopheles gambiae*, responding differently to various stresses including infection with *Plasmodium falciparum*; low levels of circulating trehalose significantly reduced parasite infection, suggesting that trehalose plays a role in the sporogonic development of the parasite (Liu et al., 2013). These results could inspire further studies on arboviral infections, by carrying out inactivation or RNAi studies targeting trehaloneogenesis proteins to find out whether viral replication and vectorial competence of *Ae. aegypti* are affected.

Finally, the additional knowledge gained could contribute to a better understanding of the pathogen blocking effect, enabling the conception of transgenic mosquito lines devoid of *Wolbachia* but having kept the pathogen-blocking effect. In addition, this knowledge will help in designing new-generation insecticides targeting trehaloneogenesis proteins, as is currently being done (García and Argüelles, 2021; Matassini et al., 2020).
